# Exploring the gut microbiota-hippocampus-metabolites axis dysregulation in sepsis mice

**DOI:** 10.3389/fmicb.2024.1302907

**Published:** 2024-05-17

**Authors:** Fangqiang Song, Qinglun Li, Jiyao Cui, Jianhua Wang, Shuai Xiao, Bo Yu, Yanqi Sun, Wenke Song, Linlin Wu, Yongqin Zhou

**Affiliations:** ^1^Department of Critical Care Medicine, Tengzhou Central People’s Hospital, Tengzhou, China; ^2^Translational Pharmaceutical Laboratory, Jining NO. 1 People’s Hospital, Jining, China; ^3^Department of Oncology, Tengzhou Central People’s Hospital, Tengzhou, China

**Keywords:** 16S ribosomal RNA, gut microbiota, hippocampus dysfunction, metabolite, sepsis, ultra-high-performance liquid chromatography tandem mass spectrometry

## Abstract

**Background:**

Sepsis is commonly associated with a sudden impairment of brain function, thus leading to significant rates of illness and mortality. The objective of this research was to integrate microbiome and metabolome to reveal the mechanism of microbiota-hippocampus-metabolites axis dysfunction in a mouse model of sepsis.

**Methods:**

A mouse model of sepsis was established via cecal ligation and puncture. The potential associations between the composition of the gut microbiota and metabolites in the hippocampus of mice with sepsis were investigated by combining 16S ribosomal RNA gene sequencing and ultra-high-performance liquid chromatography tandem mass spectrometry.

**Results:**

A total of 140 differential metabolites were identified in the hippocampal tissues of mice with sepsis when compared to those of control mice. These differential metabolites in mice with sepsis were not only associated with autophagy and serotonergic synapse, but also involved in the metabolism and synthesis of numerous amino acids. At the phylum level, the abundance of *Bacteroidota* was increased, while that of *Firmicutes* (*Bacillota*) was decreased in mice with sepsis. At the genus level, the abundance of *Alistipes* was increased, while that of *Lachnospiraceae_NK4A136_group* was decreased in mice with sepsis. The *Firmicutes* (*Bacillota*)/*Bacteroidota* (F/B) ratio was decreased in mice with sepsis when compared to that of control mice. Furthermore, the F/B ratio was positively correlated with 5′-methylthioadenosine, PC (18:3(9Z,12Z,15Z)/18:0) and curdione, and negatively correlated with indoxylsulfuric acid, corticosterone, kynurenine and ornithine.

**Conclusion:**

Analysis revealed a reduction in the F/B ratio in mice with sepsis, thus contributing to the disturbance of 5′-methylthioadenosine, curdione, PC (18:3(9Z,12Z,15Z)/18:0), corticosterone, ornithine, indoxylsulfuric acid and kynurenine; eventually, these changes led to hippocampus dysfunction. Our findings provide a new direction for the management of sepsis-induced hippocampus dysfunction.

## Introduction

Sepsis, a life-threatening syndrome, is associated with pathological, physiological, and biochemical dysfunction caused by infection (Singer et al., 2016). Any person affected by infection can potentially develop sepsis and it has been estimated that the incidence of sepsis ranges from 0.4 per 1,000 to 1 per 1,000 (Angus et al., 2001; Harrison et al., 2006). In addition, some studies have reported that the incidence of sepsis can be as high as 1–2% of all hospitalized patients and that the proportion of patients admitted to intensive care units with sepsis can be as high as 11.1% (Kaukonen et al., 2014). Although significant progress has been made in the development of anti-infection treatments over recent years, the incidence of sepsis is still increasing annually; this represents a major issue because sepsis patients still face psychological, physical and cognitive disorders, even after treatment (Iwashyna et al., 2012; Gaieski et al., 2013). In particular, cognitive disorders are prominently observed in the initial stages of sepsis; however, there is no clinical evidence to support a direct infection within the central nervous system of such cases (Iwashyna et al., 2010; Adam et al., 2013). Therefore, gaining a better understanding of the pathogenicity underlying the initiation and progression of sepsis is imperative if we are to identify novel therapies for sepsis-induced hippocampus dysfunction.

Both humans and animals evolved in intimate association with microbial communities. The gut microbiota is known to play a crucial role in organ development, metabolism and the immune system (Collins et al., 2014; Dabke et al., 2019; Zheng et al., 2020). The human body harbors an immense concentration of microorganisms within the gut microbiota. Accumulating evidence now supports the fact that the gut microbiota is strongly associated with the pathogenesis and outcome of sepsis (Dickson, 2016; Corriero et al., 2022; Wozniak et al., 2022). For example, some bacterial species, such as *Staphylococcus* and *Escherichia coli*, are known to thrive, translocate and cause sepsis in the absence of anaerobes (Adelman et al., 2020). Several bacterial metabolites, including indole-3-propionic acid and short-chain fatty acids, have been shown to influence the physiological functionality of a host (Erny et al., 2015; Hyland et al., 2022). In addition, the microbiota-gut-brain-axis describes the physiological communication link responsible for the exchange of information between microbiota in the gut and the brain (Santocchi et al., 2016). Previous studies involving germ-free mice reported the important role of the gut microbiota in maintaining cognitive function by regulating the activation of microglia, neurogenesis and myelination in the central nervous system (Erny et al., 2015; Ogbonnaya et al., 2015; Hoban et al., 2016). In addition, Fang et al. developed a mouse model of sepsis by performing cecal ligation and puncture (CLP) and subsequently demonstrated that mice with sepsis exhibited cognitive deficits (Fang et al., 2022). These authors also showed that the gut of mice with sepsis was enriched with *Enterobacteriaceae* (Fang et al., 2022). These publicly available data provide additional evidence to support the significant involvement of the microbiota-gut-brain-axis in sepsis-induced hippocampus dysfunction.

Metabolomics is increasingly being used in a wide variety of research disciplines, particularly to identify biomarkers. At present, very little is known about the metabolic alterations that occur in the hippocampal tissues during sepsis, despite an extensive body of research aiming to identify metabolic markers of sepsis in the serum and intestinal tract. Moreover, the combination of metabolomics and 16S rRNA sequencing has been utilized to analyze the composition of the gut microbiota and its correlation with metabolic phenotypes or functional alterations in different diseases, including sepsis (Stewart et al., 2017; Sun et al., 2023). Therefore, we used an integrated approach that combined ultra-high-performance liquid chromatography tandem mass spectrometry (UHPLC–MS/MS)-based metabolomics technology with 16S rRNA sequencing to investigate potential associations between components of the gut microbiota and metabolic pathways in the hippocampus. Our findings enhance our understanding of sepsis-related hippocampus dysfunction in mice and provide an appropriate foundation for the development of innovative treatment options.

## Methods

### Animals and experimental design

C57BL/6 mice, 8-weeks-of-age and weighing between 20 and 25 g, were acquired from Pengyue Experimental Animal Breeding Co., Ltd. (Jinan, China) and maintained in plastic cages in a controlled environment with a 12 h light/dark cycle at 20–22°C and unlimited access to food and water. The mice were acclimatized to the experimental conditions for one week prior to the experiments.

Following acclimatization, the mice were randomly divided into control and sepsis groups (*n* = 6 per group). Sepsis was then induced in the model mice by applying an experimental procedure, known as CLP, as described previously (Rittirsch et al., 2009). In brief, the mice were intraperitoneally administered with pentobarbital sodium (50 mg/kg) for anesthesia and subsequently moved into the supine position on an operating table for fixation. A small incision, measuring 0.4 cm, was then created along the midline of the abdomen to reveal the cecum. Next, a silk suture, 1–0 in thickness, was used to ligate the cecum at a point located 1 cm from the tip. Then, we used a 20-gage needle to create a single puncture point located 0.5 cm away from the ligation site. This was followed by gentle compression of the cecum to facilitate fecal release. Subsequently, we repositioned the bowel within the abdominal cavity and sutured the abdominal skin. Mice in the control group were subjected to identical procedures but without ligation or puncture of the cecum. Following surgery, all mice received 1 mL of 0.9% normal saline for subcutaneous resuscitation. One day after the CLP procedure, the mice were humanely euthanized with an intraperitoneal injection of pentobarbital sodium at a dosage exceeding 200 mg/kg. The hippocampal tissues were immediately harvested on ice, frozen in liquid nitrogen, and then stored at-80°C for subsequent experiments. All experimental procedures conformed to the ARRIVE guidelines (Animal Research: Reporting *in vivo* Experiments) and were approved by the Ethical Committee for Animal Experimentation of Jining No. 1 People’s Hospital (Approval Number: JNRM-2023-DW-060).

### Sample preparation

Each hippocampal tissue (50 mg) was placed into a 2 mL centrifuge tube, followed by the addition of a grinding bead with a diameter of 6 mm. For the extraction of metabolites, we used 400 μL of an extraction solution consisting of methanol and water in a ratio of 4:1 (v:v). The extraction solution also contained an internal standard (L-2-chlorophenylalanine) at a concentration of 0.02 mg/mL. The samples were then ground with a frozen tissue grinder (Wonbio-96c; Wanbo Biotechnology, Shanghai, China) for 6 min at-10°C and with a frequency of 50 Hz. Subsequently, low-temperature ultrasonic extraction was performed for 30 min at 5°C and with a frequency of 40 kHz. Samples were then stored at-20°C for 30 min and then centrifuged for 15 min at 4°C and with a speed of 13,000 g. The resulting supernatants were each transferred to an injection vial for UHPLC–MS/MS analysis. As key aspect of the quality control process, a pooled quality control sample was also prepared for analysis by combining equal volumes of all samples.

### UHPLC–MS/MS-based metabolomics analysis

Each sample was analyzed by a Thermo UHPLC-Q Exactive HF-X system equipped with an ACQUITY HSS T3 column (100 mm × 2.1 mm × 1.8 μm; Waters, Milford, MA, United States). The flow rate was set to 0.40 mL/min and the temperature of the column was maintained at 40°C. For the mobile phases, solvent A consisted of a mixture of water and acetonitrile (95:5, v/v) containing 0.1% formic acid, while solvent B contained a combination of acetonitrile, isopropanol and water (47.5:47.5, v/v) with 0.1% formic acid.

In positive ion mode, the separation gradient conditions were as follows: from 0 to 20%, mobile phase B was used for the first 3 min; then from 3–4.5 min, mobile phase B increased to reach a concentration of 35%. From 4.5–5 min, mobile phase B increased further to reach a concentration of 100%. Subsequently, mobile phase B was used alone for 1.3 min; finally, in the last two tenths of a min there was transition back to mobile phase A only.

For negative ion mode, the separation gradient conditions were slightly different. During the first 1.5 min, only up to 5% of mobile phase B was used. Between 1.5 and 2 min, this percentage increased up to 10%. Between 2 and 4.5 min, the percentage of mobile phase B continued to increase up to 30%. Between 4.5 and 5 min, we used 100% mobile phase B; this was applied for a further 1.3 min. Finally, in the last two tenths of a minute, there was transition back to mobile phase A only.

For mass spectrometry analysis, we used the Thermo UHPLC-Q Exactive HF-X Mass Spectrometer (MS) which was equipped with an electrospray ionization source that operated in both positive and negative modes. The optimal experimental conditions included a source temperature of 425°C, an ion-spray voltage at 3500 V (positive mode) and-3500 V (negative mode), a sheath gas flow rate of 50 arb, an Aux gas flow rate of 13 arb, and a normalized collision energy ranging from 20 to 40 to 60 V for UHPLC–MS/MS. The UHPLC–MS/MS resolution was set to 7,500, while the full MS resolution reached up to 60,000. Data acquisition followed the data dependent acquisition mode within a mass range spanning from 70 to1050 m/z during detection.

### Metabolomic data and pathway enrichment analysis

Next, we used Progenesis QI software (Waters Corporation, Milford, United States) to preprocess the raw UHPLC–MS/MS data. Subsequently, a CSV-formatted three-dimensional data matrix was exported. This three-dimensional matrix included mass spectral response intensity, metabolite name, and sample information. Next, we removed internal standard peaks and false positive peaks from the peak pooled and data matrix. Metabolites were then identified using the Majorbio Database, Metlin^1^ and HMDB.^2^

The “ropls” package in R (Version 1.6.2) was then used to perform orthogonal least partial squares discriminant analysis (OPLS-DA). Compounds with variable importance in projection (VIP) values ≥1.0, *p* < 0.05 and a |log2(Foldchange)| ≥ 0 were considered as potential differential expressed metabolites between the control and sepsis groups. Enrichment analysis was conducted using the Kyoto Encyclopedia of Genes and Genomes (KEGG) database.^3^

### 16S rRNA sequencing of the gut microbiota

An E.Z.N.A. stool DNA isolation Kit (Omega Bio-tek, Norcross, GA, U.S.) was used to extract microbial genomic DNA from colonic contents; this DNA was then separated by 1% agarose gel electrophoresis. The V3–V4 regions of the bacterial 16S rRNA gene were then amplified by TransStart Fastpfu DNA Polymerase (TransGen AP221-02) on a PCR system (Gene Amp 9,700, ABI, USA). PCR was isolated and purified before sequencing with a TruSeqTM DNA Sample Prep Kit (Illumina, USA) on an Illumina MiSeq platform (Illumina, USA). Raw data was acquired by applying the RS_ReadsOfinsert1 protocol. High-quality sequences were then acquired using the QIIME package.^4^ Cluster analysis was performed for operational taxonomic units (OTUs) in UPARSE (version 7.1) with a 97% similarity cutoff. Chimeric sequences were identified and eliminated from further analysis. Next, the Ribosomal Database Program Classifier Bayesian Algorithm was used to conduct species taxonomic analyses against the Silva 16S rRNA database at a confidence threshold of 70%. This was followed by beta diversity analysis and community composition analysis. Finally, a Spearman’s correlation heatmap was generated to evaluate potential associations between the contents of the gut microbiota and metabolites.

### Enzyme-linked immunosorbent assay

The mouse corticosterone ELISA Kit was obtained from Meibiao Biotechnology (Yancheng, China). Mouse ornithine ELISA Kit, mouse 5′-methylthioadenosine ELISA Kit and mouse curdione ELISA Kit were purchased from Meimian Industrial Co., Ltd. (Yancheng, China). The concentrations of corticosterone, ornithine, 5′-methylthioadenosine and curdione in hippocampus were measured through ELISA using the corresponding commercial Kits.

### Statistical analysis

Differences in 16S ribosomal RNA gene sequencing were gaged by applying Wilcoxon rank-sum test. The variations among metabonomics data were analyzed by Student’s test. Pearson’s correlation analysis was used to identify correlations. Statistical significance was set at *p* < 0.05.

## Results

### Clear differences were observed between the control and sepsis groups

**Figure 1:**
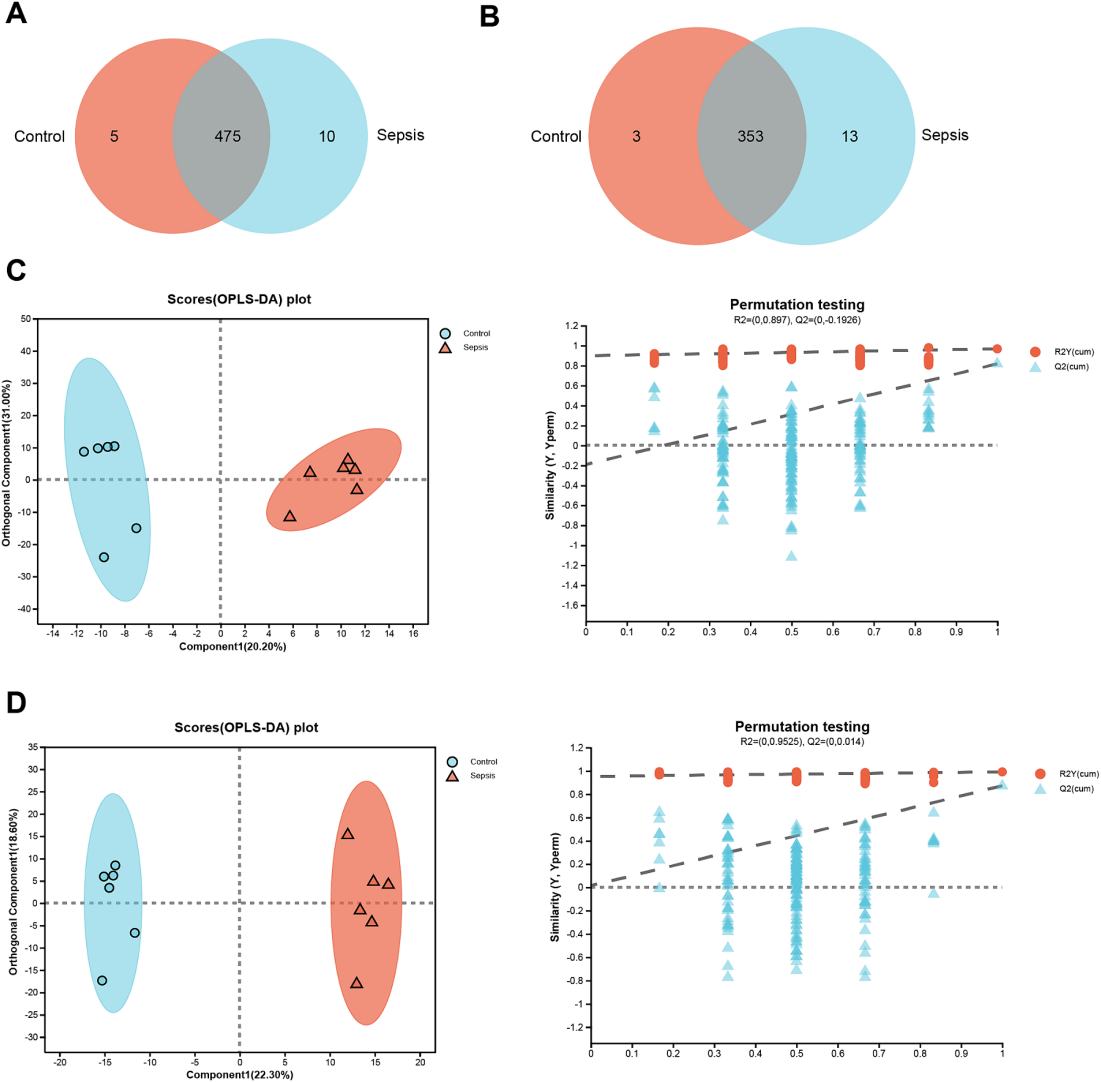
Clear differences are observed between control and sepsis samples. **(A)** Venn diagram of the metabolites between the control and sepsis groups in positive ion mode. **(B)** Venn diagram of the metabolites between the control and sepsis groups in negative ion mode. **(C)** OPLS-DA score plots and 200 permutation tests in positive ion mode. **(D)** OPLS-DA score plots and 200 permutation tests in negative ion mode.

In positive ion mode, a total of 475 common metabolites were identified between the control and sepsis hippocampus samples (Figure 1A); only 353 metabolites were identified between the control and sepsis hippocampus samples in negative ion mode (Figure 1B). Furthermore, OPLS-DA models showed that in positive ion mode, there were clear differences in the expression profiles of metabolites between control and sepsis samples (Figure 1C). In addition, the intersection points between the blue regression line (Q^2^-point) and vertical axis were all negative values (Figure 1C), thus indicating that the OPLS-DA models and the prediction were reliable. Similar patterns were observed under negative ion mode (Figure 1D).

### Differential analysis of metabolomic expression profiles

**Figure 2:**
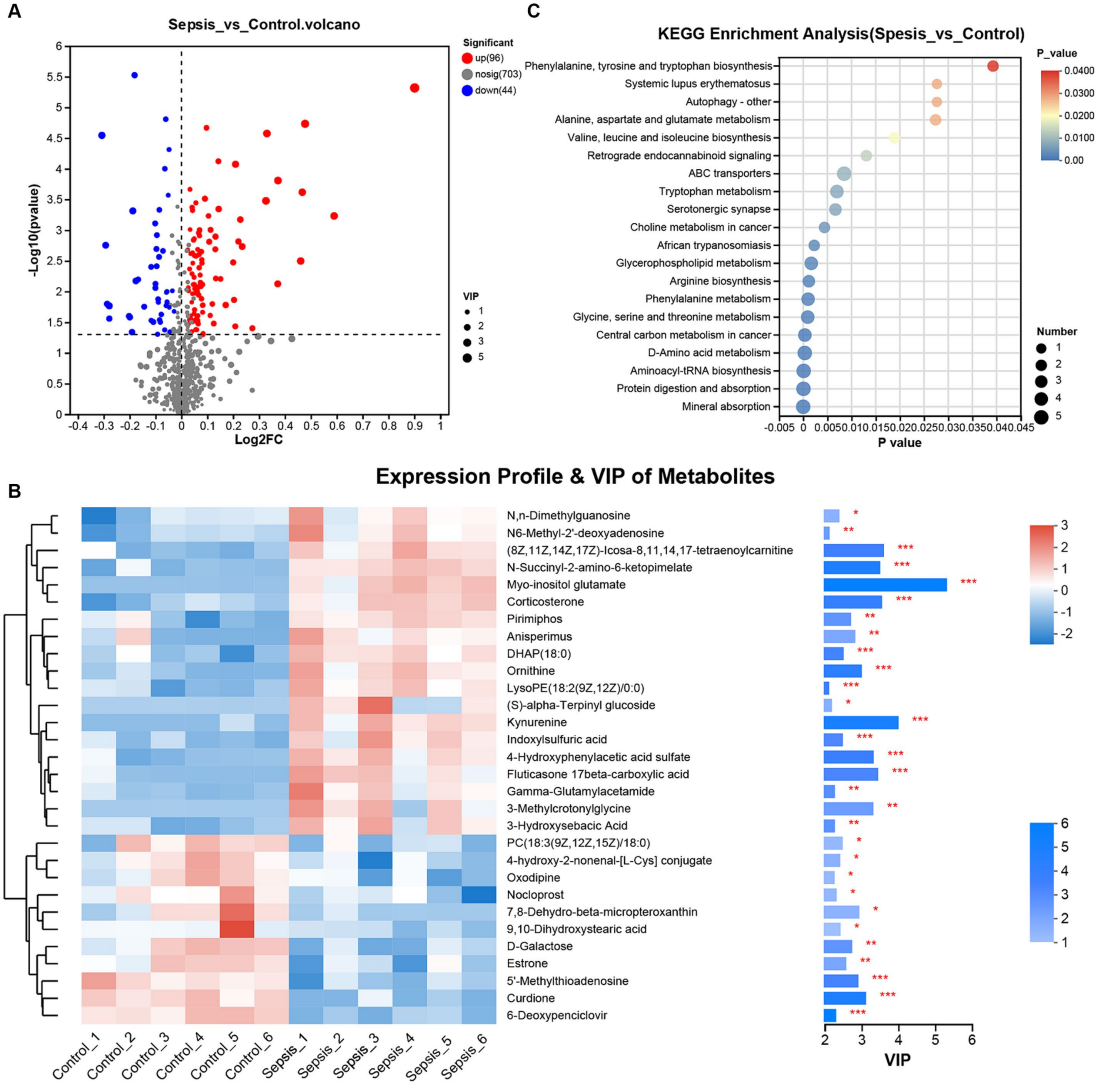
Differential analysis for metabolomic expression profiles. **(A)** The volcano plot of differentially expressed metabolites between the control and sepsis groups (Student’s t test). **(B)** Heatmap and VIP value bar chart of differentially expressed metabolites between the control and sepsis groups (Pearson’s correlation analysis). **(C)** KEGG was used for enrichment analysis of the differentially expressed metabolites between the control and sepsis groups. ^*^p < 0.05, ^**^p < 0.01, ^***^p < 0.001.

Generation of a volcano plot (VIP ≥ 1.0, *p* < 0.05 and |log2(Foldchange)| ≥ 0) of the differentially expressed metabolites between the control and sepsis samples identified 140 differentially expressed metabolites (96 upregulated and 44 downregulated) in the sepsis samples (Figure 2A). Next, we generated a heatmap by performing cluster analysis of the differentially expressed metabolites between the control group and the sepsis group (Figure 2B). Analysis showed that the CLP procedure induced the upregulation of several metabolites, including N,N-dimethylguanosine, N6-Methyl-2′-deoxyadenosine, (8Z,11Z,14Z,17Z)-Icosa-8,11,14,17-tetraenoylcarnitine, N-Succinyl-2-amino-6-ketopimelate, myo-inositol glutamate, corticosterone, pirimiphos, anisperimus, DHAP (18:0), ornithine, LysoPE [18:2(9Z,12Z)/0:0], (S)-alpha-Terpinyl glucoside, kynurenine, indoxylsulfuric acid, 4-hydroxyphenylacetic acid sulfate, fluticasone 17beta-carboxylic acid, gamma-glutamylacetamide, 3-methylcrotonylglycine and 3-hydroxysebacic acid. However, CLP also induced downregulation of other metabolites, including PC [18:3(9Z,12Z,15Z)/18:0], 4-hydroxy-2-nonenal-[L-Cys] conjugate, oxodipine, nocloprost, 7,8-dehydro-beta-micropteroxanthin, 9,10-dihydroxystearic acid, D-galactose, estrone, 5′-methylthioadenosine, curdione and 6-deoxypenciclovir.

KEGG enrichment analysis further indicated that the differentially expressed metabolites in mice with sepsis mice were not only associated with autophagy and serotonergic synapse, but also involved in amino acid biosynthesis (phenylalanine, tyrosine, tryptophan, valine, leucine, isoleucine, arginine) and metabolism (alanine, aspartate, glutamate, tryptophan, phenylalanine glycine, serine and threonine) (Figure 2C).

Next, we determined the abundance of two nerve-injury-related metabolites (corticosterone and ornithine) and two neurotrophy-related metabolites (5′-methylthioadenosine and curdione). Compared to the control group, the abundances of corticosterone and ornithine were significantly higher in the sepsis group (Supplementary Figure S1A; *p* < 0.001), while the abundances of 5′-methylthioadenosine and curdione were significantly decreased (Supplementary Figure S1B; *p* < 0.001). Additionally, the concentrations of these four identified metabolites in hippocampus tissues were also validated using ELISA. As illustrated in Supplementary Figures S1C, D, it was shown that compared to the control mice, the concentrations of corticosterone and ornithine in the hippocampus tissues of sepsis mice were increased (*p* < 0.001), whereas both 5′-methylthioadenosine and curdione concentrations were decreased (*p* < 0.001). These results were consistent with the data analyzed by metabolomics.

### Alterations of the gut microbiota in mice with sepsis

**Figure 3:**
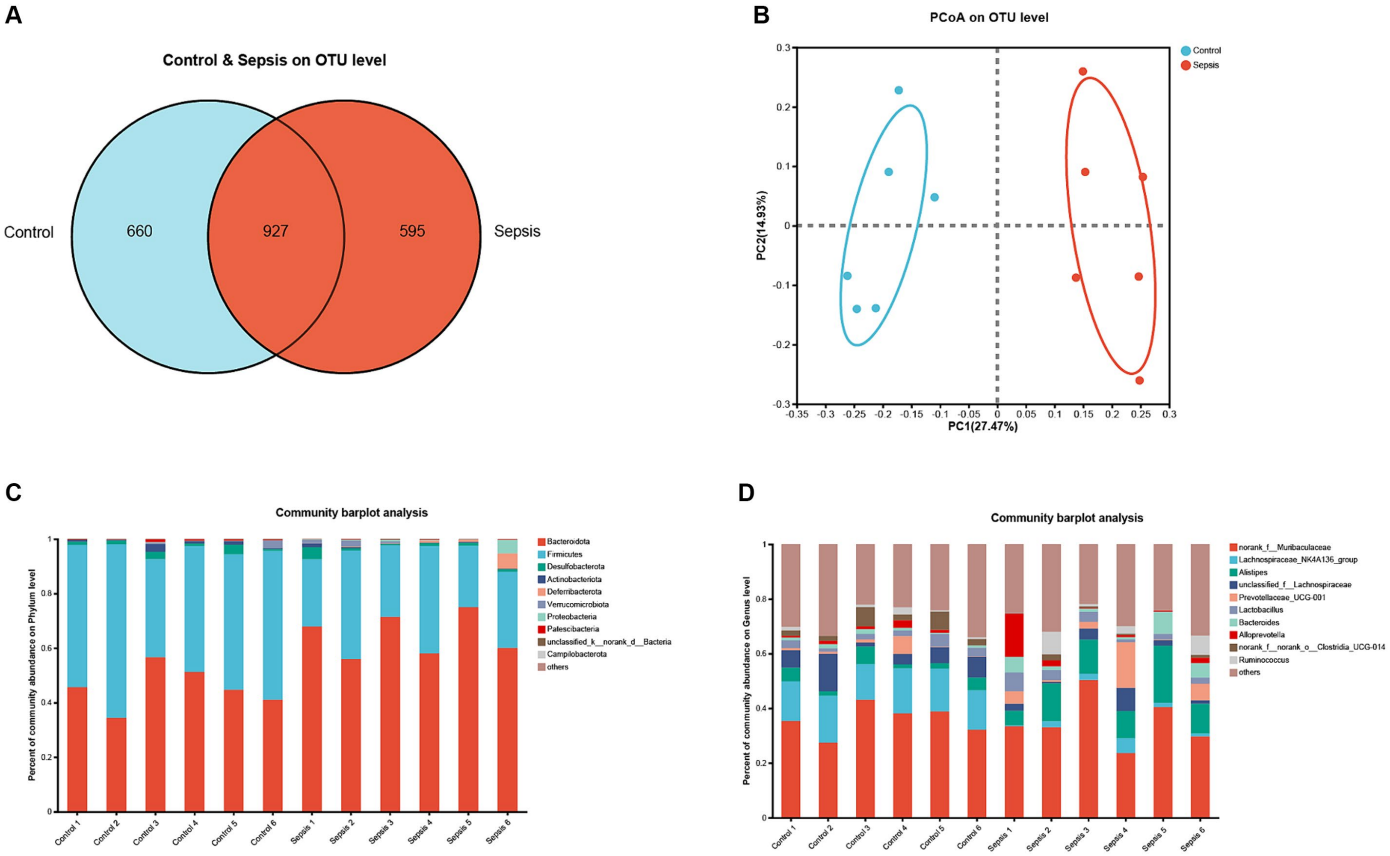
The changes of gut microbiota in sepsis mice. **(A)** OTU distribution Venn diagram. **(B)** PCoA on OUT level between the control and sepsis groups. **(C)** Histogram of gut microflora at the phylum level. **(D)** Histogram of gut microflora at the genus level.

Next, we analyzed the gut microbiota in mice with sepsis at the OTU level. As shown in Figure 3A, a total of 927 OTUs were present in all samples. Furthermore, analysis showed that the control group exhibited a greater species abundance than the sepsis group. Next, principal coordinate analysis (PCoA) was used to analyze beta diversity (Figure 3B); in this figure, the horizontal axis corresponds to the first principal component, while the vertical axis corresponds to the second principal component. Analysis showed that the first principal component contributed 27.47% to the sample difference; this compared to 14.93% for the second principal component. At the OUT level, PCoA revealed a significant separation between the control group and the sepsis group. Further analysis of bacterial communities revealed that the gut microbiota of the control group primarily consisted of *Bacteroidota*, *Firmicutes* (*Bacillota*), *Desulfobacterota* and *Actinobacteriota* at the phylum level; this compared to *Bacteroidota*, *Firmicutes* (*Bacillota*), *Deferribacterota*, *Verrucomicrobiota*, *Proteobacteria* and *Campilobacterota* in the sepsis group (Figure 3C). At the genus level, the gut microbiota in the control group was mainly composed of *norank_f__Muribaculaceae*, *Lachnospiraceae_NK4A136_group*, *Alistipes, unclassified_f__Lachnospiraceae*, *Prevotellaceae_UCG-001*, *Lactobacillus*, *Bacteroides* and *norank_f__norank_o__Clostridia_UCG-014*, while *norank_f__Muribaculaceae*, *Lachnospiraceae_NK4A136_group*, *Alistipes, unclassified_f__Lachnospiraceae*, *Prevotellaceae_UCG-001*, *Lactobacillus*, *Bacteroides, Alloprevotella* and *Ruminococcus* (Figure 3D).

Next, we used the Wilcoxon rank-sum test to compare populations of different bacterial species between the control and sepsis groups. As illustrated in Supplementary Figure S2A, at the phylum level, mice in the sepsis group had significantly higher proportions of *Bacteroidota* (*p* < 0.01), *Deferribacterota* (*p* < 0.01), *Proteobacteria* (*p* < 0.01) and *Campilobacterota* (*p* < 0.05), but lower proportions of *Firmicutes* (*Bacillota*) than those in the control group (*p* < 0.05). Compared to the control group, the proportion of *Bacteroidota* in the sepsis group was significantly increased (*p* < 0.01) while the proportion of *Firmicutes* (*Bacillota*) was decreased (*p* < 0.05; Supplementary Figure S2B). As illustrated in Supplementary Figure S2C, at the genus level, the Wilcoxon rank-sum test revealed that mice in the sepsis group had significantly higher proportions of *Alistipes* (*p* < 0.01), *Oscillibacter* (*p* < 0.05), *Rikenellaceae_RC9_gut_group* (*p* < 0.01), Mucispirillum (*p* < 0.01), *Eubacterium_nodatum_group* (*p* < 0.01), *norank_f__norank_o__Clostridia_vadinBB60_group* (*p* < 0.01), *Parabacteroides* (*p* < 0.01) and *Bilophila* (*p* < 0.05), but significantly lower proportions of *Lachnospiraceae_NK4A136_group* (*p* < 0.01), *norank_f__norank_o__Clostridia_UCG-014* (*p* < 0.05), *norank_f__Lachnospiraceae* (*p* < 0.01), *Lachnoclostridium* (*p* < 0.01), *norank_f__norank_o__RF39* (*p* < 0.05), *norank_f__Erysipelotrichaceae* (*p* < 0.05) and *unclassified_f__Ruminococcaceae* (*p* < 0.05) than those in the control group. Compared to the control group, the proportion of *Alistipes* was significantly increased (*p* < 0.01) in the sepsis group while the proportion of *Lachnospiraceae_NK4A136_group* was significantly decreased (*p* < 0.01; Supplementary Figure S2D).

### Relevance analysis between metabolites and the gut microbiota

**Figure 4:**
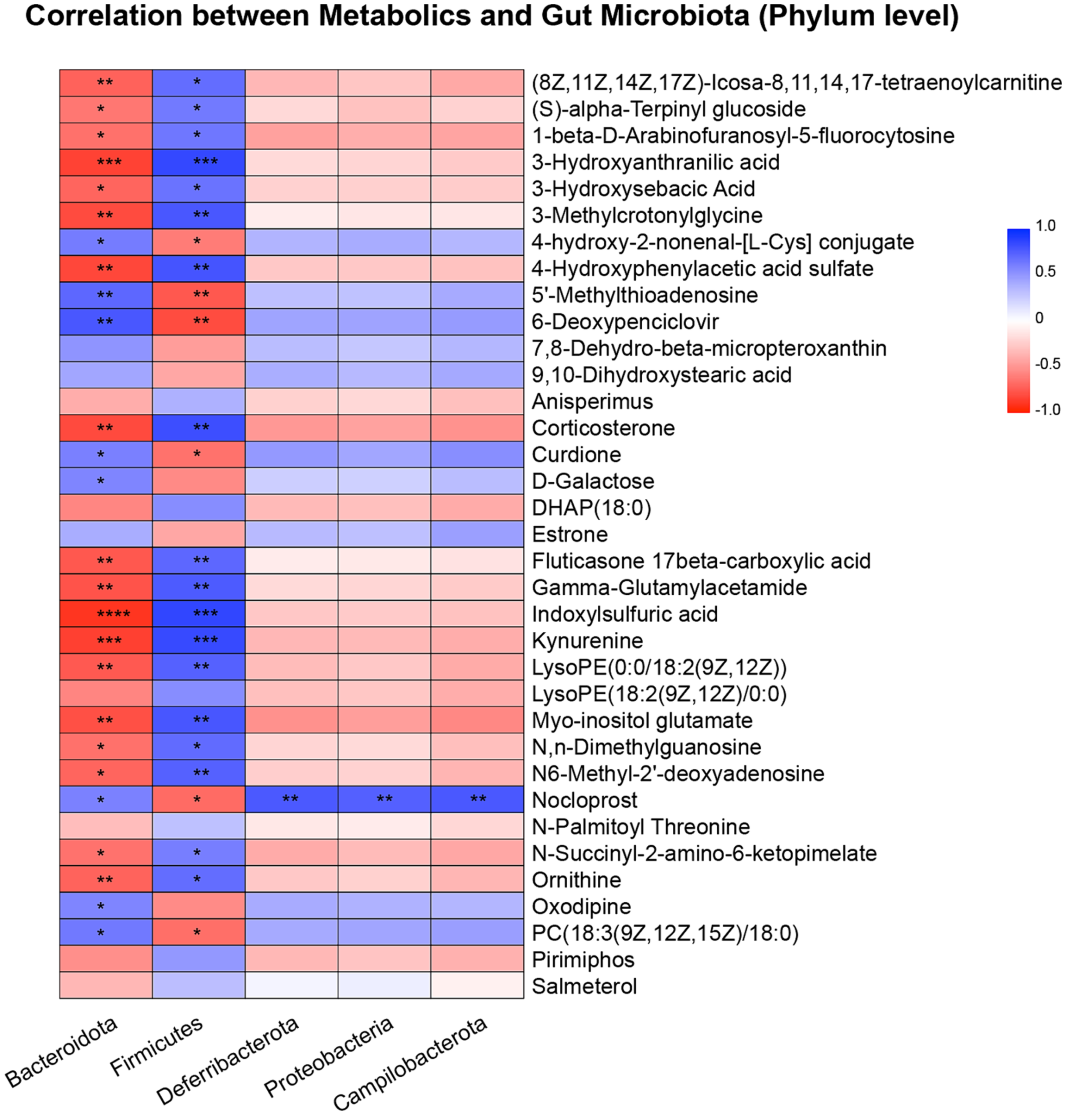
Pearson correlation heatmap of metabolites and gut microbiota at the phylum level. ^*^p < 0.05, ^**^p < 0.01, ^***^p < 0.001, ^****^p < 0.0001.

Next, we investigated correlations between hippocampal metabolites and the gut microbiota. As shown in Figure 4, we found that *Bacteroidota* exhibited strong correlations with various metabolites. For example, *Bacteroidota* was positively correlated with (8Z,11Z,14Z,17Z)-Icosa-8,11,14,17-tetraenoylcarnitine, (S)-alpha-Terpinyl glucoside, 1-beta-D-Arabinofuranosyl-5-fluorocytosine, 3-hydroxyanthranilic acid, 3-hydroxysebacic Acid, 3-methylcrotonylglycine, 4-hydroxyphenylacetic acid sulfate, corticosterone, fluticasone 17beta-carboxylic acid, gamma-glutamylacetamide, indoxylsulfuric acid, kynurenine, LysoPE (0:0/18:2(9Z,12Z)), myo-inositol glutamate, N,N-dimethylguanosine, N6-Methyl-2′-deoxyadenosine, N-Succinyl-2-amino-6-ketopimelate and ornithine, while was negatively correlated with 4-hydroxy-2-nonenal-[L-Cys] conjugate, 5′-methylthioadenosine, 6-deoxypenciclovir, curdione, nocloprost and PC [18:3(9Z,12Z,15Z)/18:0]. In contrast, the *Firmicutes* (*Bacillota*) phylum exhibited opposite results.

### The firmicutes (Bacillota)/Bacteroidota (F/B) ratio Was correlated with metabolites

**Figure 5:**
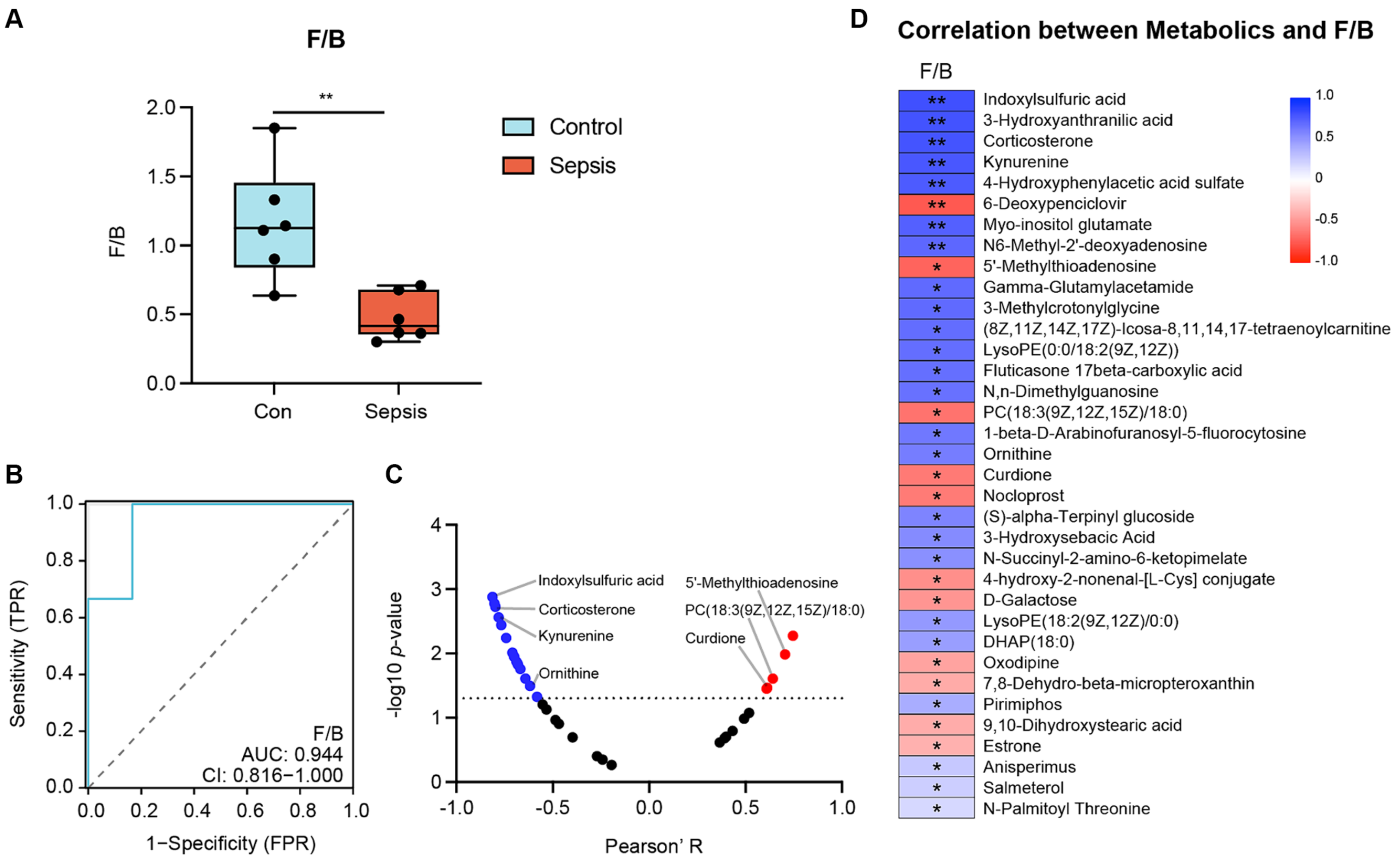
The correlation between *Firmicutes* (*Bacillota*)/*Bacteroidota* (F/B) ratio and metabolites. **(A)** F/B ratio between the control and sepsis groups (Student’s t test). **(B)** Receiver Operating Characteristic (ROC) Curve demonstrated that F/B ratio can distinguish the sepsis mice from the control mice (AUC = 0.944). **(C)** Correlation analysis of F/B ratio with metabolites (Pearson’s correlation analysis). **(D)** Heatmap of the correlation analysis of F/B ratio with differential metabolites (Pearson’s correlation analysis). ^*^p < 0.05, ^**^p < 0.01.

Next, we investigated the F/B ratio in controls and mice with sepsis. As illustrated in Figure 5A, the F/B ratio was significantly lower in the sepsis group than in the control group (*p* < 0.01). Receiver Operating Characteristic (ROC) curve analysis further demonstrated that the F/B ratio was able to distinguish mice with sepsis from controls (AUC = 0.944), thus indicating that the F/B ratio may play a key role in the progression of sepsis (Figure 5B). A scatter diagram was generated to visualize the correlation between F/B ratio and differential metabolites (Figure 5C). Furthermore, a heatmap was created in the scatter diagram, to identify significant differential metabolites (Figure 5D). Analysis revealed that the F/B ratio was positively correlated with 5′-methylthioadenosine, PC (18:3(9Z,12Z,15Z)/18:0) and curdione (Supplementary Figure S3A), but negatively correlated with indoxylsulfuric acid, corticosterone, kynurenine and ornithine (Supplementary Figure S3B).

## Discussion

Sepsis is generally complicated by the dysfunction of multiple organs, especially the brain. Previous research has shown that hippocampus dysfunction is predominantly associated with various mediators released during sepsis, and involves alterations in awareness, including disorientation to delirium or even a state of unconsciousness (Chung et al., 2020). The presence of cerebral dysfunction during sepsis is known to contribute to prolonged stays in the intensive care unit and higher mortality rates among patients with sepsis (Sonneville et al., 2017). Research has also demonstrated that the main pathophysiological features of sepsis-induced hippocampus dysfunction are inflammatory responses, oxidative stress and neuronal loss in the hippocampus (Barichello et al., 2007; Semmler et al., 2007; Imamura et al., 2011). However, prior to the present study, the metabolic signature in the hippocampus and the potential association between metabolic alterations in the hippocampus and changes in the gut microbiome during the progression of sepsis was poorly understood.

*Lachnospiraceae* possesses the capability to convert plant polysaccharides into short-chain fatty acids, such as butyric acid, via the process of fermentation (Boutard et al., 2014). The *Lachnospiraceae_NK4A136_group* genus, belonging to the family of *Lachnospiraceae*, is considered as a potential producer of butyrate to treat dementia (Dou et al., 2020; Stadlbauer et al., 2020). Several investigations have reported that butyrate exhibited neuroprotective properties in various models of neurological conditions, including depression, cognitive decline, and neurodegenerative diseases (Stilling et al., 2016). The *Alistipes* genus is a relatively recent sub-branch genus of the *Bacteroidota* phylum (Parker et al., 2020). Some recent studies have indicated that the abundance of *Alistipes* is significantly increased in multiple mental and brain diseases including anxiety, depression and autism spectrum disorder (Bangsgaard Bendtsen et al., 2012; Jiang et al., 2015; Strati et al., 2017). Collectively, these previous data suggested that in healthy individuals, the *Lachnospiraceae_NK4A136_group* should be dominant in the intestines, along with a reduced abundance of *Alistipes*. In the present study, we found that the abundance of the *Lachnospiraceae_NK4A136_group* was significantly reduced in mice with sepsis when compared to mice in the control group. In addition, the abundance of *Alistipes* was increased in mice from the sepsis group, thus concurring with previous reports. Therefore, we speculated that increasing the community numbers of butyrate-producing bacteria and inhibiting the *Alistipes* community may be promising therapeutic options for treating sepsis-induced hippocampus dysfunction. Future experimental studies should investigate this hypothesis. Additionally, it is acknowledged that gut microbiota is predominantly composed of bacteria but contains other commensals such as archaea, viruses, fungi, and protozoa (Feng et al., 2018). However, in some nervous system injury-related diseases such as Parkinson’s disease, the results regarding fungi, archaea, and viruses were excluded because only two genera (*Nitrososphaera* and *Methanobrevibacter*) from archaea were reported in three of the eligible articles (Lin et al., 2018; Qian et al., 2018; Li et al., 2019), and only a single paper focused on fungi without any report of a difference between the Parkinson’s disease group and the healthy control group (Cirstea et al., 2021). Similarly, in this study, the differences of fungi, archaea, and viruses were not observed in the gut of control mice and sepsis mice. Of course, this does not mean that these microorganisms are not related to sepsis-induced hippocampus dysfunction. We will conduct more detailed research in the future.

Brain tissues exhibit increased susceptibility to oxidative stress when compared to other types of tissue due to their lower levels of antioxidants, higher peroxidizable lipid levels, and increased oxygen consumption rate (Andersen, 2004; Jankord and Herman, 2008; Zafir and Banu, 2009a). Therefore, some factors that induce oxidative stress may lead to damage to the brain tissues. Some studies have demonstrated that an increased level of corticosterone is associated with oxidative stress, thus leading to damage in the central nervous system (McEwen, 2007; Zafir and Banu, 2009b). In addition, ornithine, a metabolite of arginine in the urea cycle, has been shown to induce oxidative stress and reduce the integrity of the blood–brain barrier in a rat model of acute pancreatitis (Walter et al., 2022). In the present study, we found that the abundances of corticosterone and ornithine were significantly increased in the hippocampus of mice with sepsis when compared to that of control mice. These results implied that sepsis may induce alterations in the levels of corticosterone and ornithine in the hippocampus and may cause oxidative stress damage in the brain tissues.

5′-methylthioadenosine is a metabolite generated from S-adenosylmethionine during the synthesis of spermidine and polyamines spermine, thus acting as an inhibitor of polyamine biosynthesis (Evans et al., 2004). The therapeutic potential of 5′-methylthioadenosine was first reported in experimental models of liver cancer and hepatic injury (Latasa et al., 2010). More recently, the neuroprotective effects of 5′-methylthioadenosine have been also reported (Moreno et al., 2014). Curdione is one of the active constituents of *Curcuma zedoaria* and is known to exhibit anti-inflammatory, anti-cancer and antimicrobial properties (Lai et al., 2004; Makabe et al., 2006; Li et al., 2014). Recently, Li et al. demonstrated that curdione also exhibited pharmacological neuroprotective activity against cerebral ischemia reperfusion injury in rats (Li et al., 2017). These data also suggested that both 5′-methylthioadenosine and curdione act as neurotrophic factors to protect against nerve injury. In the present study, we observed reduced levels of 5′-methylthioadenosine and curdione in mice with sepsis when compared to control mice. These results implied that CLP procedure led to reductions in the levels of key neurotrophic factors, including 5′-methylthioadenosine and curdione, thus accelerating the dysfunction of brain tissues.

The *Firmicutes* (*Bacillota*) and *Bacteroidota* phyla are known to represent the major components of the gut microbiota. The F/B ratio has been considered as a relevant marker of gut dysbiosis in human diseases; for example, obese animals and humans are known to exhibit a higher F/B ratio when compared to individuals of normal weight (Magne et al., 2020). More importantly, *Firmicutes* (*Bacillota*) was identified as a neuroprotective factor in some hippocampus dysfunction-related diseases, including Alzheimer’s disease, autism spectrum disorder, and major depressive disorders (Finegold et al., 2010; Jiang et al., 2015; McEvoy et al., 2017). Previous research showed that these diseases were associated with the dominance of *Bacteroidota* in the intestines, concurrent with a reduction in *Firmicutes* (*Bacillota*) (Finegold et al., 2010; Yu et al., 2017; Merra et al., 2020). In other words, in such cases, there was a reduction in the F/B ratio, thus generating gut dysbiosis and hippocampus dysfunction. Therefore, we hypothesized that similar alterations in the abundances of *Firmicutes* (*Bacillota*) and *Bacteroidota* lead to hippocampus dysfunction in mice with sepsis. In the present study, we found that the abundance of *Bacteroidota* was increased in mice with sepsis, with a concurrent reduction in *Firmicutes* (*Bacillota*). Thus, the F/B ratio was significantly reduced in mice with sepsis. These results indicated that sepsis could lead to gut dysbiosis via a reduction in F/B ratio.

Next, we investigated the correlation between F/B ratio and hippocampal metabolites. F/B ratio was positively correlated with 5′-methylthioadenosine, curdione and PC [18:3(9Z,12Z,15Z)/18:0], and negatively correlated with corticosterone, ornithine, indoxylsulfuric acid and kynurenine. Except for these oxidative stress-related metabolites (corticosterone and ornithine) and neurotrophic factors (5′-methylthioadenosine and curdione), indoxylsulfuric acid and kynurenine are known to be related to inflammation (Tan et al., 2021; Willette et al., 2021), while PC [18:3(9Z,12Z,15Z)/18:0] has been reported to mediate intestinal metabolic balance (Kennelly et al., 2018). A recent commentary, published by Gareau et al., highlighted the important role of the microbiota-gut-brain axis in sepsis-associated hippocampus dysfunction (Gareau, 2022). Microorganisms can mediate gut-brain signaling by inducing the production of neurotransmitters and metabolites, which can also generate certain neuroactive compounds themselves (Morais et al., 2021). Therefore, we further concluded that sepsis may mediate signaling involving the microbiota-gut-brain axis signaling by disrupting key metabolites in the hippocampus, including 5′-methylthioadenosine, curdione, PC [18:3(9Z,12Z,15Z)/18:0], corticosterone, ornithine, indoxylsulfuric acid and kynurenine, eventually inducing the onset of hippocampus dysfunction.

The potential limitations of this study should not be disregarded. First, for more exact identification, further genetic techniques, such as metagenomics and metaproteomics may be required. Second, the analysis of correlation alone is insufficient without delving into the underlying mechanism, necessitating further experimental validation. Third, it is only analyzed in animal models and requires clinical sample validation.

## Conclusion

In this study, we found that sepsis led to changes in the gut microbiota and hippocampal metabolomic pathways in mice model. We also found that the CLP procedure disrupted the levels of corticosterone, ornithine, 5′-methylthioadenosine and curdione in the hippocampus. In addition, we found that sepsis reduced the F/B ratio, and that a reduced F/B ratio was strongly associated with the disruption of 5′-methylthioadenosine, curdione, PC [18:3(9Z, 12Z, 15Z)/18:0], corticosterone, ornithine, indoxylsulfuric acid and kynurenine. These factors may form part of the regulatory mechanism for sepsis-induced hippocampus dysfunction.

## Data availability statement

The metabolomics data presented in the study are deposited in the metabolights repository, accession number MTBLS9869. The 16S rRNA data presented in the study are deposited in the NCBI BioProject repository, accession number PRJNA1096837.

## Ethics statement

The animal study was approved by the Ethical Committee for Animal Experimentation of Jining No.1 People’s Hospital (Approval No. JNRM-2023-DW-060). The study was conducted in accordance with the local legislation and institutional requirements.

## Author contributions

FS: Conceptualization, Methodology, Writing – review & editing, Data curation, Formal analysis, Investigation, Project administration, Resources, Software, Supervision, Validation, Visualization, Writing – original draft, Funding acquisition. QL: Formal analysis, Methodology, Software, Visualization, Writing – review & editing. JC: Formal analysis, Methodology, Software, Visualization, Writing – review & editing. JW: Data curation, Formal analysis, Writing – review & editing. SX: Formal analysis, Methodology, Software, Visualization, Writing – review & editing. BY: Formal analysis, Methodology, Software, Visualization, Writing – review & editing. YS: Formal analysis, Methodology, Software, Visualization, Writing – review & editing. WS: Formal analysis, Methodology, Software, Visualization, Writing – review & editing. LW: Conceptualization, Data curation, Project administration, Resources, Supervision, Validation, Writing – review & editing. YZ: Conceptualization, Data curation, Project administration, Resources, Supervision, Validation, Writing – review & editing.

## Footnotes

^1^ https://metlin.scripps.edu/

^2^ http://www.hmdb.ca/

^3^ http://www.genome.jp/kegg/

^4^Version 1.9.1, http://qiime.org/install/index.html
