# Supplementary figures and images for: Exploring the gut microbiota-hippocampus-metabolites axis dysregulation in sepsis mice
Source: Front Microbiol. 2024 May 17;15:1302907. doi: 10.3389/fmicb.2024.1302907 (PMC11140095; doi:10.3389/fmicb.2024.1302907)

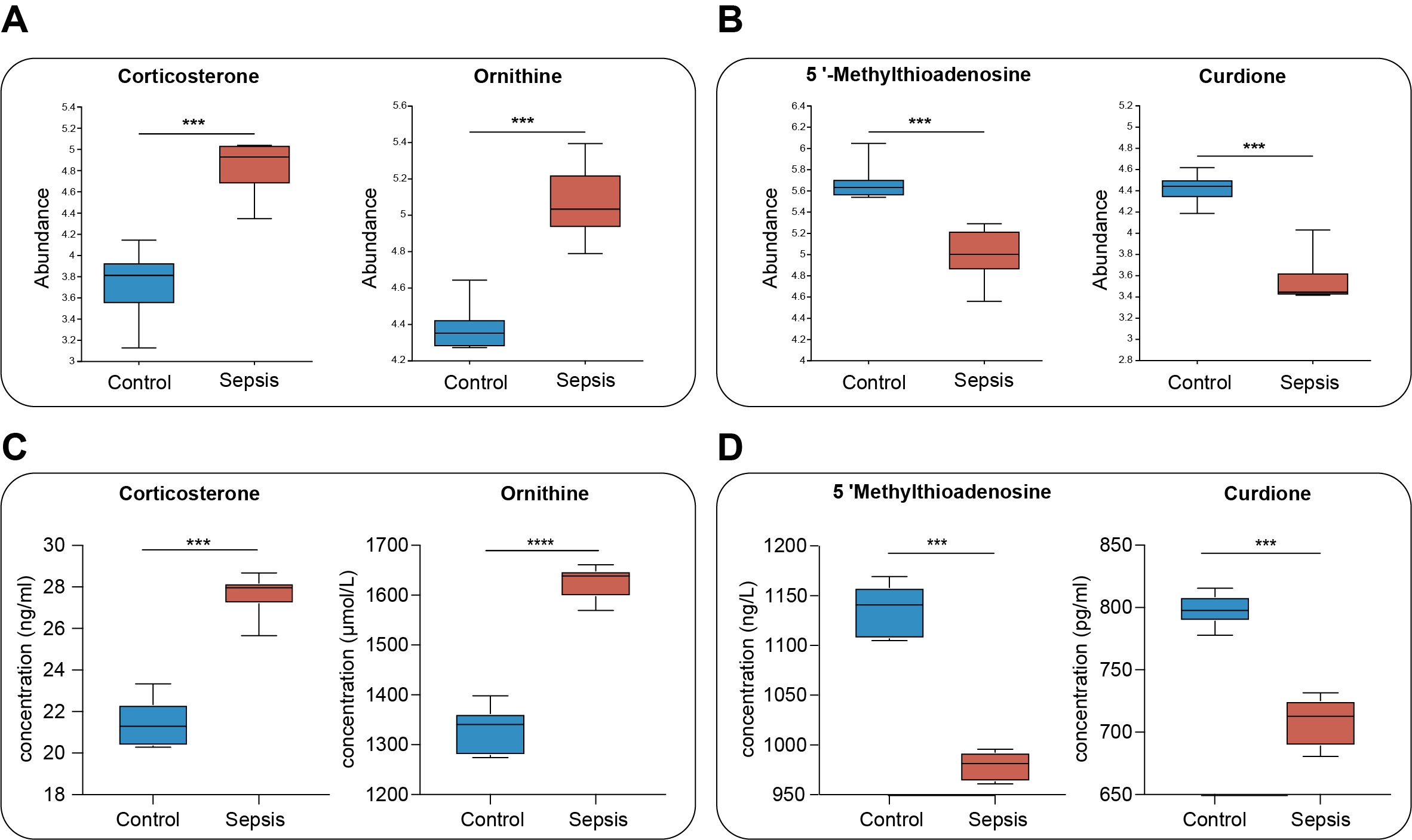

Supplement: Supplementary file 1 [file Image_1.TIF]

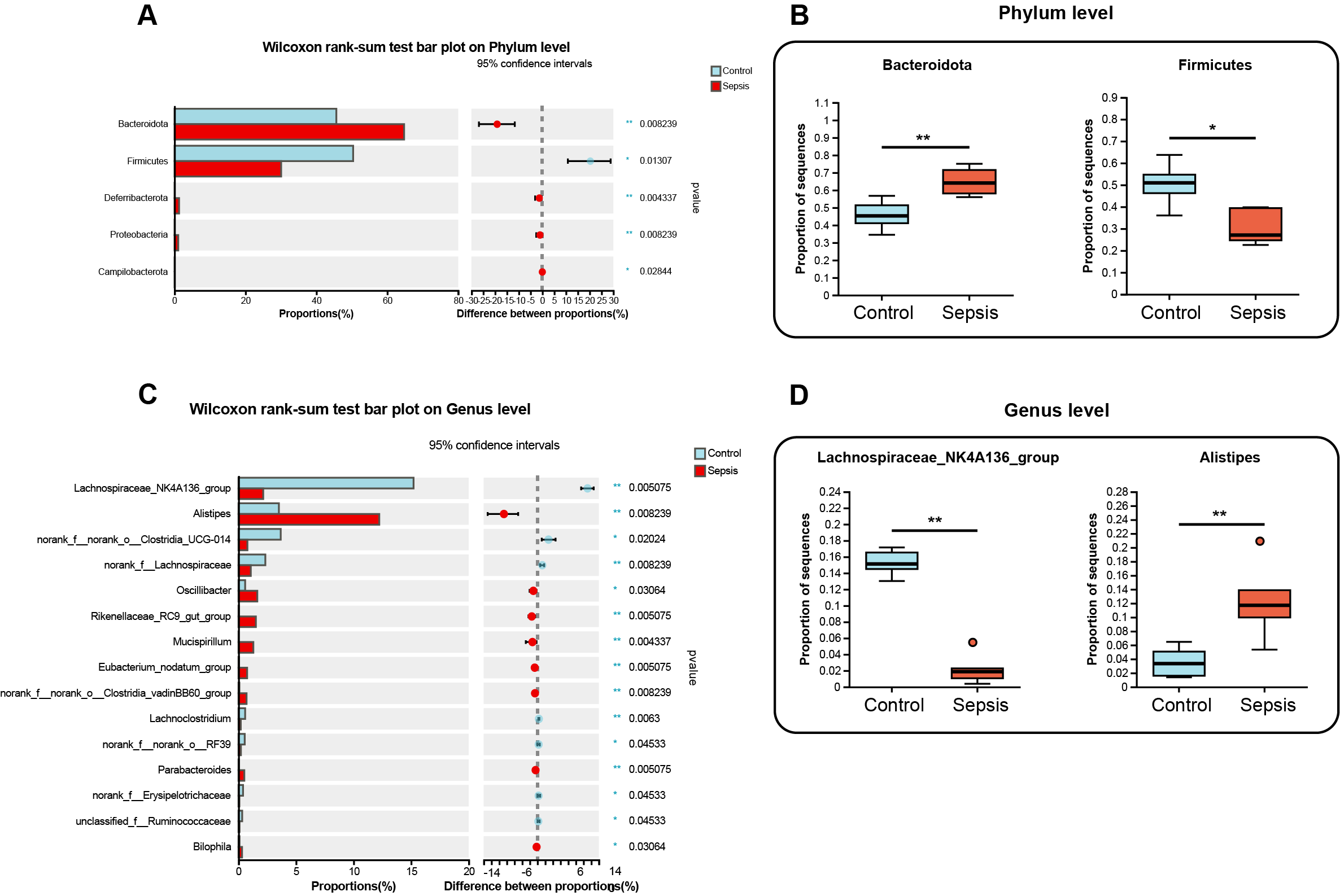

Supplement: Supplementary file 2 [file Image_2.TIF]

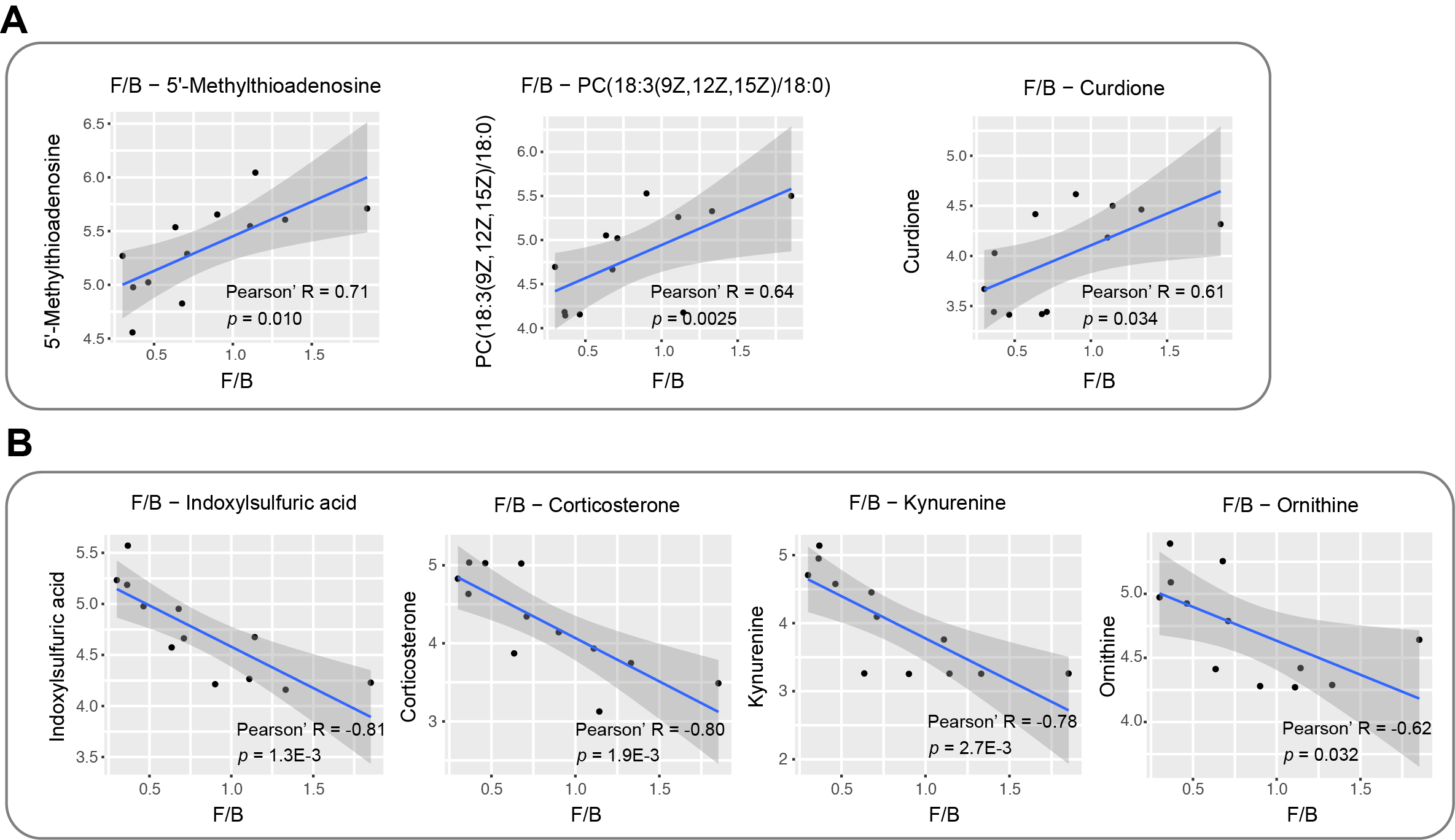

Supplement: Supplementary file 3 [file Image_3.TIF]
